# Effects of ursolic acid on muscle mass and bone microstructure in rats with casting-induced muscle atrophy

**DOI:** 10.20463/jenb.2019.0022

**Published:** 2019-09-30

**Authors:** Yun Seok Kang, Eun Bi Noh, Sang Hyun Kim

**Affiliations:** 1. Department of Sports Science, Chonbuk National University, Jeollabuk-do Republic of Korea

**Keywords:** ursolic acid, skeletal muscle, bone mineral density, bone microstructure, exercise mimetics

## Abstract

**[Purpose]:**

Recent studies suggest that ursolic acid (UA) is a potential candidate for a resistance exercise mimetic that can increase muscle mass and alleviate the deleterious effect of skeletal muscle atrophy on bone health. However, these studies evaluated the effects of UA on skeletal muscle and bone tissues, and they have not verified whether such effect could occur concurrently on muscle and bone, as is the case with resistance exercise. Thus, the aim of this study was to analyze the effect of UA injection on muscle mass and bone microstructure using an animal model of atrophy to demonstrate the potential of UA as a resistance exercise mimetic.

**[Methods]:**

The immobilization (IM) method was used on the left hindlimb of Sprague Dawley (SD) rats for 10 days to induce muscle atrophy, whereas the right hindlimb was used as an internal control (IC). The animal models were divided into two groups, SED (sedentary, n=6) and UA (n=6) to demonstrate the effect of UA on atrophic skeletal muscles. The UA group received a daily intraperitoneal injection of UA (5 mg/kg/day) for 8 weeks. After 10 days of IM, the data collected for the IC were compared with that of IM to determine whether muscle atrophy might occur.

**[Results]:**

Muscle atrophy was induced and bone mineral density (BMD) decreased significantly. The 8-week UA treatment significantly increased the gastrocnemius muscle mass compared to the SED group. In regard to the effect of UA on bones, negative results such as a decrease in BMD, trabecular bone volume fraction, and trabecular number, and an increase in trabecular separation, were observed in the SED group, but no such difference was observed in the UA group. No significant difference was observed in atrophic hindlimbs between SED and UA groups.

**[Conclusion]:**

These results alone are insufficient to suggest that UA is a potential resistance exercise mimetic for atrophic skeletal muscle and weakened bone. However, this study will help determine the potential of UA as a resistance exercise mimetic.

## INTRODUCTION

“If we had a pill that contained all the benefits of exercise, it would be the most widely prescribed drug in the world,” stated Ronald M. Davis, former president of the American Medical Association. Recently, efforts to search for an exercise pill has increased^1^^,^^2^. An exercise pill contains bioactive compounds that mimic the beneficial effects of exercise without exercising^3^. Major efforts have focused on approaches to understand the molecular mechanisms underlying fatty acid oxidation, mitochondrial biogenesis, and oxidative fiber-type transformation induced by activation of peroxisome proliferator-activated receptor gamma coactivator-1α to determine whether compounds such as AICAR, GW1516, and resveratrol mimic the health effects of aerobic exercise^4^. According to a systematic review by Katashimaet al.,^5^ ursolic acid (UA) showed the same beneficial effects of aerobic and resistance exercises on sarcopenia and obesity.

UA is a pentacyclic triterpenoid present in many plants such as rosemary, basil fruit, apple, and coffee. It has a range of biological activities^4^^,^^6^. In regard to muscle mass, UA has beneficial effects similar to those of resistance exercise. UA improves insulin/insulin-like growth factor-1 (IGF-1) signaling, reduces atrophy by decreasing the expression of muscle RING-finger protein-1 (MuRF1) and muscle atrophy F-box (MAFbx)^7^, and induces muscle hypertrophy^7^^-^^10^. The effects of UA treatment when used alone have not been demonstrated in an animal model of atrophy with hindlimb immobilization (IM), but UA in combination with other treatments such as low-intensity treadmill exercise is effective in increasing skeletal muscle mass^11^. If UA shows beneficial effects similar to those exhibited by resistance exercise, it can be proposed as an exercise pill for the prevention of osteoporosis because resistance exercise is one of the best methods to strengthen bones^12^. Beginning with a study reporting that UA increases bone-forming activity^13^, several studies report that UA inhibits osteoclast production^14^^,^^15^ and bone loss^16^^,^^17^. However, no research has been conducted on the potential effects of UA as a resistance exercise mimetic that simultaneously ameliorates muscle atrophy and the deleterious effect on bones.

In this study, the effects of dietary UA on muscle mass and bone microstructure were investigated using a Sprague-Dawley (SD) rat model of atrophy. The aim of this study was to demonstrate the potential of UA as a resistance exercise mimetic.

## METHODS

This study used 18 8-week-old male Sprague-Dawley (SD) rats (Central Lab. Animal Inc., Seoul, Korea). Following an adaptation period of 1 week in a laboratory setting with constant temperature (21℃) and humidity (40–60%), IM was performed on the left hindlimb of SD rats to induce muscle atrophy and the right hindlimb was used as an internal control (IC). After a 10 day IM period, six rats were euthanized and examined for IM-induced atrophy and the corresponding levels of bone mineral density (BMD). SD rats were divided into two groups after immobilization, SED (sedentary, n=6) and UA (n=6) groups, and treated with UA for the following 8 weeks. Body weight was measured using an electronic balance (Navigator N0B110; Ohaus Co., NJ, USA) once a week between 9 and 10 am before UA injection, and the skeletal muscles and bones were extracted from the euthanized rats after 8 weeks of treatment for analysis. Rats were euthanized by cervical dislocation under anesthesia. All rats were allowed free access to food (carbohydrate 58.9%, fat 12.4%, protein 28.7%; Purina corp. MO, USA) and water without restrictions.

### Casting-induced muscle atrophy and UA injection

Rats were anesthetized with pentobarbital sodium (50 mg/kg of body mass; Fort Dodge Animal Health, USA) and immobilized using plaster casts^18^. Rats had plaster casts on the left hindlimb only and the right hindlimb was reserved for use as IC, thus no treatment was given. During the IM period, rats were monitored daily for clinical conditions of the hindlimb such as edema and discoloration, and SD rats with such issues were excluded from the experiment.

UA treatment was carried out as per the same method used in a previous study^11^. UA was dissolved in distilled water containing 0.1% Tween 80 (Sigma, USA) and administered by intraperitoneal injection (5 mg/kg) once per day (Liu et al., 2013). The SED group received the same dose of vehicle (distilled water containing 0.1% Tween 80) by intraperitoneal injection.

### Tissue collection and muscle mass

Tissue collection was performed under anesthesia using pentobarbital sodium (50 mg/kg of body mass; Fort Dodge Animal Health, USA). Skeletal muscles such as gastrocnemius (GAS), soleus (SOL), tibialis anterior (TA), and extensor digitorum longus (EDL) were extracted and weighed using an electronic balance (EPG213, Ohaus Co., USA). The weight measurements were used to calculate the recovery rate for the IC.

### Bone microstructure

After skeletal muscles were collected, the hip joint area was cut and fixed in 10% paraformaldehyde, followed by micro-CT (SkyScan 1076 system; Bruker microCT, Kontich, Belgium) imaging taken for bone microstructure assessment. 

For the cancellous bone of proximal tibia, CT Analyzer software (Bruker microCT, Kontich) was used to analyze the BMD, trabecular bone volume fraction (BV/TV), trabecular number (Tb.N), and trabecular separation (Tb.Sp) within the region of interest (ROI) of the proximal growth plate. Three-dimensional (3D) images were generated using CT Vol Realistic Visualization software (Bruker microCT, Kontich).

### 5. Statistical analysis

Data are presented as mean ± standard error of the mean (SEM) using SPSS 12.0 (SPSS Inc., Chicago, Illinois, USA). An unpaired t-test was conducted to compare skeletal muscle mass during the 10-day IM, and a one-way ANOVA was carried out to evaluate the improvements in skeletal muscle mass after 8 weeks of UA treatment. In addition, two-way ANOVA was used to determine the bone-related effects of 8-week UA treatment between the treatment conditions (SED and UA) and internal control (IC) or immobilization (IM). A Bonferroni post hoc test was performed, and the significance level was p<0.05.

## RESULTS

### Changes in muscle mass and BMD during the 10-day IM 

**Figure 1.**
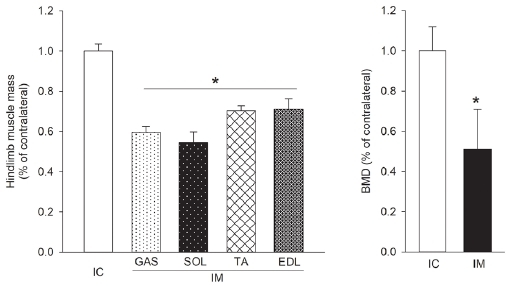
Ten-day hindlimb immobilization decreased muscle mass and BMD. IC, internal control; IM, immobilization; GAS, gastrocnemius; SOL, soleus; TA, tibialis anterior; EDL, extensor digitorum longus; BMD, bone mineral density. *p<0.05 vs. IC by t-test. N=3 rats.

The effects of IM-induced hindlimb muscle atrophy on BMD were examined to evaluate the beneficial effects of UA treatment on muscle mass and the deleterious effect on bones caused by IM (Fig 1). Ten days of hindlimb IM resulted in decreased muscle mass for GAS (41%), SOL (45%), TA (30%), and EDL (29%) muscles (*p*<0.05). BMD was reduced by 49% (*p*<0.05).

### Effects of UA on atrophic skeletal muscle and the deleterious effect on bones

**Figure 2.**
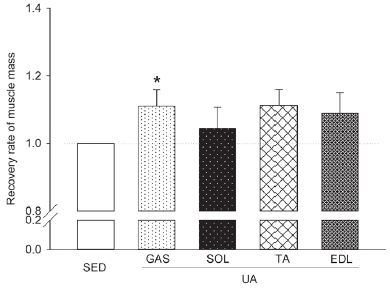
Recovery rate of skeletal muscle mass after 8 weeks of UA treatment. SED, sedentary group; UA, ursolic acid injection group; GAS, gastrocnemius; SOL, soleus; TA, tibialis anterior; EDL, extensor digitorum longus.*p<0.05 vs. IC by t-test. N=6 rats.

Fig 2 shows the recovery rate of muscle mass for the UA group, with the SED group mean set at 100%. The 8-week UA treatment showed a recovery rate varying from 4.5% to 11.3% depending on muscle type. However, a significant increase (*p*<0.05) was only observed in the GAS muscle.

**Figure 3.**
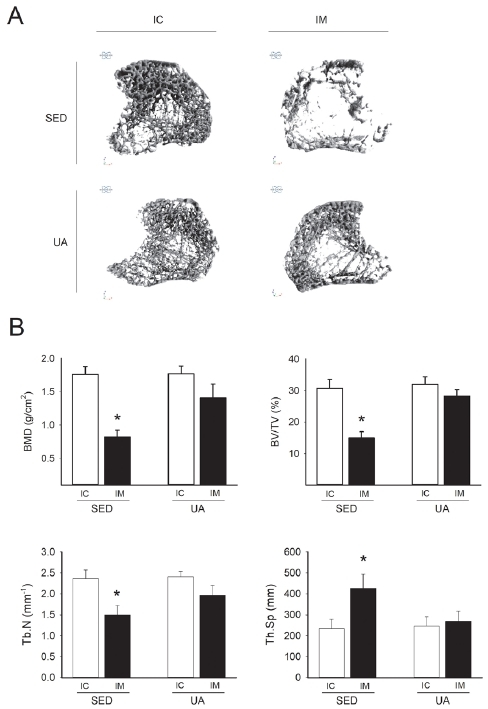
Changes in BMD and bone microstructure after 8 weeks of UA treatment. (A) Typical 3D images of proximal tibia metaphysis in each group. (B) Metaphyseal trabecular bone parameters of the proximal tibia in each group. SED, sedentary group; UA, ursolic acid injection group; IC, internal control; IM, immobilization; GAS, gastrocnemius; SOL, soleus; TA, tibialis anterior; EDL, extensor digitorum longus; BMD, bone mineral density; BV/TV, metaphyseal trabecular bone volume fraction; Th.N, trabecular number; Tb. Sp, trabecular separation. *p<0.05 vs. IC by two-way ANOVA. N=6 rats.

Fig 3 summarizes the changes in BMD and bone microstructure observed by micro-CT. In the SED group, BMD, BV/TV, Tb.N, and Th.Sp showed negative results (*p*<0.05) due to IM, but no statistical difference was observed between IC and IM concerning the 8-week UA treatment (Fig. 3B). These results were also observed in 3D images (Fig 3A). However, there was no statistically significant difference between the SED and UA groups regarding IM for all categories.

## DISCUSSION

The aim of this study was to demonstrate the beneficial effects of UA on muscle mass and bone microstructure, rather than explore concrete UA mechanisms for alleviating the deleterious effects of skeletal muscle atrophy on the bone.

Skeletal muscle-bone interaction involves crosstalk^19^^-^^21^. Therefore, resistance exercise for building muscle can be a highly effective treatment to preserve and increase BMD and bone strength^22^^,^^23^. However, due to the high risk of musculoskeletal injury^24^, resistance exercise has limited applications, especially in the elderly with less physical strength. The discovery of a substance that mimics the effect of resistance exercise, allowing users to achieve the positive effects of high-intensity resistance exercise with low-intensity exercise, will benefit many individuals who require resistance exercise, such as the one designed to alleviate the deleterious effect of muscle atrophy on the bone.

UA reduces atrophy by inhibiting the expression of hypertrophy- and atrophy-related genes MuRF1 and MAFbx, and increasing the activity of the insulin/IGF-1 signaling pathway^7^^,^^8^^,^^10^. Recent studies show that UA prevents retinoic acid-induced bone loss^16^ and ameliorates the deleterious effect on bones in streptozotocin-induced diabetic mice^17^. Such beneficial effects of UA involve bone formation induced by the regulated expression of osteoblast-specific genes, such as mitogen-activated protein kinases, nuclear factor NF-κB, and activator protein-1^13^. These effects are based on enhanced osteogenesis caused by NF-κB signaling^15^, tryptophan hydroxylase 1^25^, and suppressed osteoclast differentiation. These effects of UA indicate potential as a bioactive compound that mimics the beneficial effects of resistance exercise on the bone. However, these discoveries alone are insufficient for UA to be accepted as a resistance exercise mimetic. To date, there have been no studies that determine whether UA can simultaneously decrease atrophy and alleviate the deleterious effects of atrophy on the bone, as is the case with resistance exercise. This study offers key findings in support of the suggestion that UA has potential as a resistance exercise mimetic. First, the effects of 8 weeks of UA treatment on atrophic skeletal muscles induced by hindlimb IM (Fig 1) were examined and an increase in GAS muscle mass was observed but no definite effects were observed in the other muscles (Fig 2). In regard to the deleterious effect of atrophy on the bone, no significant difference was observed in IM between the SED group and UA group; thus, no clear results were obtained (Fig 3). In the SED group, deterioration of BMD and bone microstructure was observed in IM to a more significant extent than in IC, whereas no significant difference was observed in the UA group, which demonstrated the potential benefits of UA on the deleterious effect on bones (Fig 3). 

In summary, this study showed that UA mimicked the beneficial effects of resistance exercise, but this discovery alone is inadequate for presenting UA as a resistance exercise mimetic. However, analysis of the relevant tissue pathways, which were not covered in this study, in combination with other modified conditions such as UA treatment period and dose, will allow a more definite conclusion to be drawn. Moreover, such a conclusion will be of great assistance in demonstrating that UA has potential as a resistance exercise mimetic for those who require resistance exercise but have limited capabilities.
